# Chronic Kidney Disease Increases Atrial Fibrillation Inducibility: Involvement of Inflammation, Atrial Fibrosis, and Connexins

**DOI:** 10.3389/fphys.2018.01726

**Published:** 2018-12-04

**Authors:** Huiliang Qiu, Chunlan Ji, Wei Liu, Yuchi Wu, Zhaoyu Lu, Qizhan Lin, Zheng Xue, Xusheng Liu, Huanlin Wu, Wei Jiang, Chuan Zou

**Affiliations:** ^1^Second Clinical Medical College, Guangzhou University of Chinese Medicine, Guangzhou, China; ^2^Department of Cardiology, Guangdong Provincial Hospital of Chinese Medicine, Guangzhou, China; ^3^Department of Nephrology, Guangdong Provincial Hospital of Chinese Medicine, Guangzhou, China; ^4^Department of Cardiology, Guangzhou Hospital of Traditional Chinese Medicine, Guangzhou, China; ^5^Department of internal medicine, Beijing University of Chinese Medicine, Beijing, China

**Keywords:** chronic kidney disease, atrial fibrillation, fibrosis, transforming growth factor-β1, inflammasome, connexins

## Abstract

Chronic kidney disease (CKD) causes atrial structural remodeling and subsequently increases the incidence of atrial fibrillation (AF). Atrial connexins and inflammatory responses may be involved in this remodeling process. In this study, nephrectomy was used to produce the CKD rat model. Three months post-nephrectomy, cardiac structure, function and AF vulnerability were quantified using echocardiography and electrophysiology methods. The left atrial tissue was tested for quantification of fibrosis and inflammation, and for the distribution and expression of connexin (Cx) 40 and Cx43. An echocardiography showed that CKD resulted in the left atrial enlargement and left ventricular hypertrophy, but had no functional changes. CKD caused a significant increase in the AF inducible rate (91.11% in CKD group vs. 6.67% in sham group, *P* < 0.001) and the AF duration [107 (0–770) s in CKD vs. 0 (0–70) s in sham, *P* < 0.001] with prolonged P-wave duration. CKD induced severe interstitial fibrosis, activated the transforming growth factor-β1/Smad2/3 pathway with a massive extracellular matrix deposition of collagen type I and α-smooth muscle actin, and matured the NLR (nucleotide-binding domain leucine-rich repeat-containing receptor) pyrin domain-containing protein 3 (NLRP3) inflammasome with an inflammatory cascade response. CKD resulted in an increase in non-phosphorylated-Cx43, a decrease in Cx40 and phosphorylated-Cx43, and lateralized the distribution of Cx40 and Cx43 proteins with upregulations of Rac-1, connective tissue growth factor and N-cadherin. These findings implicate the transforming growth factor-β1/Smad2/3, the NLRP3 inflammasome and the connexins as potential mediators of increased AF vulnerability in CKD.

**GRAPHICAL ABSTRACT:**
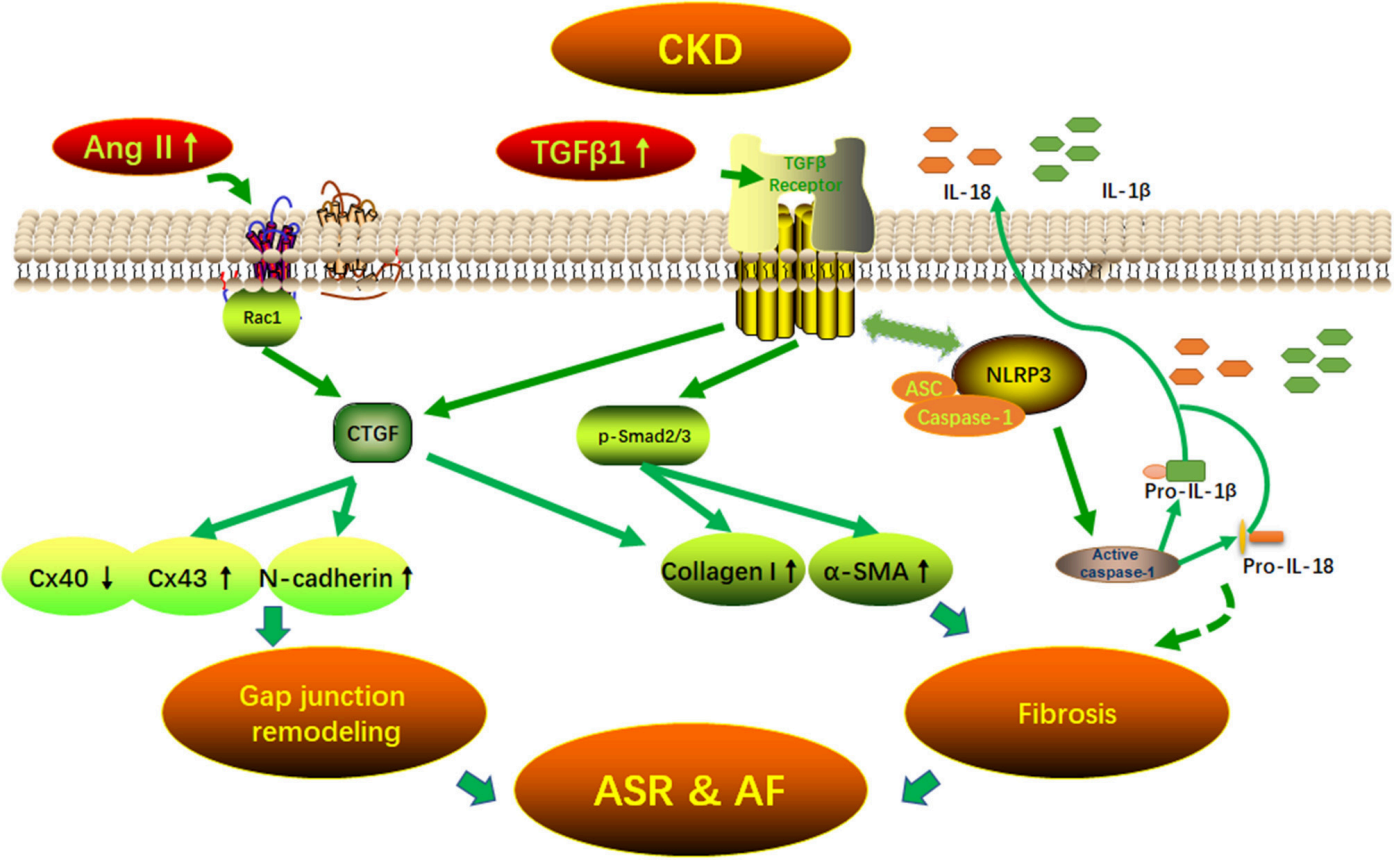
Potential mechanisms of AF arrhythmogenic substrate in CKD. Fibrosis and (connexins) gap junction remodeling are the main pathological substrate in AF development. Our findings suggest that CKD induced-atrial fibrosis may be connected with the activation of TGFβ1/Smads and the NLRP3 inflammasome signaling pathways and the CKD induced-Cx40/43 gap junction remodeling may be connected with Ang II induced-the activation of Rac-1, CTGFand N-cadherin in the atrium. AF, atrial fibrillation; Ang II, angiotensin II; ASC, apoptosis-associated speck-like protein containing a carboxy-terminal CARD; ASR, atrial structural remodeling; α-SMA, α-smooth muscle actin; Collagen I, collagen type I; CTGF, connective tissue growth factor; Cx43, connexin43; IL-1β, interleukin-1β; IL-18, interleukin-18; NLRP3, NLR (nucleotide-binding domain leucine-rich repeat-containing receptor) pyrin domain-containing protein 3; Rac1, Ras-related C3 botulinum toxin substrate 1; TGFβ1, transforming growth factor-β1.

## Introduction

Atrial fibrillation (AF) is the most common arrhythmia and increases the risk of a stroke and heart failure (Kirchhof et al., 2016). Certain factors may increase the risk of AF occurrence. These factors include age, hypertension, heart disease, and other chronic conditions such as chronic kidney disease (CKD) (Soliman et al., 2010; Liao et al., 2015; Kirchhof et al., 2016). The occurrence of AF gradually increases with progressive declines of kidney function (Olesen et al., 2012; Ng et al., 2013; Jun et al., 2015; Gill et al., 2017). The incidence of AF is 6% in elderly people without CKD, 18~21% in patients with non-dialysis-dependent CKD, and 27% in CKD patients on dialysis (Olesen et al., 2012; Ng et al., 2013; Jun et al., 2015; Gill et al., 2017). Although AF is very frequent in patients with CKD and has gained much attention as a risk factor for stroke and cardiovascular events, the mechanistic pathogenesis of AF in CKD is relatively unexplored.

Atrial structural remodeling (ASR) mainly refers to atrial enlargement and interstitial fibrosis. ASR can result in heterogeneous electrical conduction by forming reentrant activity and is one of the major pathophysiological sources of AF (Everett and Olgin, 2007). Inflammation plays an important role in the development of ASR and AF, partly by stimulating the production of angiotensin II (Ang II), while Ang II can activate inflammatory signaling which in turn acts as a pro-fibrotic factor (Corradi et al., 2008). The transforming growth factor-β1 (TGFβ1) is the main interrelated pathway involved in ASR and in the formation of atrial fibrosis in several disease conditions, including myocardial infarction, heart failure, and hypertension with upregulated Rennin-Angiotensin-Aldosterone System (RAAS) (Hao et al., 2000; Everett and Olgin, 2007; Tsai et al., 2008). TGFβ1 has been reported in the deposition of extracellular matrix through the downstream mediator Smad proteins and pro-fibrotic mediator connective tissue growth factor (CTGF) (Rosin et al., 2013). Recent studies indicate that CKD could cause atrial enlargement and fibrosis, invoke the inducible AF, and is linked to the activation of TGFβ1 (Aoki et al., 2015). However, the mediators linking TGFβ1 to fibrosis have not yet been investigated in CKD. Because of the central role of TGFβ1 in fibrogenesis, it is important to identify its downstream molecules in the atrium for future research and new therapeutic strategies.

NLR (nucleotide-binding domain leucine-rich repeat-containing receptor) pyrin domain-containing protein 3 (NLRP3) inflammasome, is one of the most well-known inflammasomes and widely participates in various immune and cellular death pathways (Gong et al., 2016). It can catalyze the pro-casepase-1 into matured caspase-1 (P10 and P20) and the latter can further mediate the pro-interleukin (IL)-1β and pro-IL-18 into the proinflammatory cytokines of IL-1β and IL-18. The NLRP3 inflammasome may play a crucial role in the development of fibrotic remodeling in organs including the kidney and heart, and can be activated in CKD mice (Gong et al., 2016; Chin et al., 2017). In addition, a Cardiomyocyte (CM)-specific knock-in a murine model (CKI, expressing functional NLRP3 inflammasome in CMs only) developed 100% premature atrial contractions with significantly increased inducible AF, whereas a selective inflammasome inhibitor MCC950 could successfully decrease the inducible AF in CKI mice (Yao et al., 2018), recognizing the NLRP3 inflammasome as a new promotor of fibrogenesis and AF. These studies clearly indicated the vital role of the NLRP3 inflammasome in the pathogenesis of fibrosis and showed that CKD is involved in the activation of the NLRP3 inflammasome in multiple organs. However, it is unclear whether the NLRP3 inflammasome can also be activated in the atrium under the condition of CKD.

ASR not only contributed to the reentrant activity but also influenced the connexins (Giepmans, 2004). Connexins abundantly distributes at the intercalated disk form the cell-to-cell electric conduction propagation that ensures the normal heart rhythm. Connexin (Cx) 40 and Cx43 are two of the mainly functional gap junction subunits in the atria (Ryu et al., 2007; Sawaya et al., 2007; Duffy and Wit, 2008). Any alterations in atrial connexins expression, phosphorylation and distribution are considered proarrhythmogenic. A previous study has demonstrated that the gap junction protein can be restructured by the activation of RAAS and CKD induced the activation of RAAS. Adam et al suggested that an increased Ang II can change the expression of Cx43 by the activation of Rac1 and nicotinamide adenine dinucleotide phosphate (NADPH) oxidase and eventually lead to atrial structural remodeling and AF (Adam et al., 2010). As NADPH oxidase has been implicated as a critical target for the pathogenesis of atrial fibrosis and the inducible AF in the CKD animal model (Fukunaga et al., 2012; Aoki et al., 2015), speculations that Cx43 can also be affected by CKD have been disregarded (Qiu et al., 2018). However, Cx40 must be highlighted, especially as it is one of the most important gap junction proteins in the atrium. The aim of the present study was to investigate the potentially mechanistic pathogenesis in the setting of CKD. We showed that TGFβ1/Smad2/3/CTGF, NLRP3 inflammasome and connexins are potential mediators of increased AF vulnerability in the setting of CKD.

## Methods

### Animals and 5/6 Nephrectomy

Animal experiments in this study were approved under the supervision of the Animal Care Committee of Guangdong Provincial Hospital of Chinese Medicine following the Regulations of Experimental Animal Administrations issued by the State Committee of Science and Technology of the People's Republic of China. Specific pathogen-free grade Sprague Dawley rats (6 weeks old, body weight: 200 ± 10 g) were provided by the Guangdong Provincial Medicinal Laboratory Animal Center [SCXK (Yue) 2013-0002, Guangzhou, China] and housed in a SPF environment. The 5/6 nephrectomy was performed to produce the CKD rat model. Animals were randomly assigned to sham-operated group (Sham, *n* = 10) and 5/6 nephrectomy-induced CKD group (CKD, *n* = 15). 5/6 nephrectomy was performed by the surgical resection of the upper and lower thirds of the left kidney, followed by whole right nephrectomy 7 days later, as previously described (Zeng et al., 2016). Rats in the sham group underwent the same procedure, without nephrectomy. Three months post-surgery, echocardiography, echocardiographic assessment, electrophysiology and AF vulnerability studies, biochemical detection, histology, immunohistochemistry, and atrial proteins expressions were performed as a follow up.

### Echocardiographic Assessment

The Visual Sonics Vevo 2100 system (VisualSonics Inc., Toronto, Canada) was used to assess cardiac function and the structure at the baseline, 3 months after surgery, as described previously (Qiu et al., 2018a,b). Animals were anesthetized with isoflurane (2%). The anterior chest was shaved carefully using an electric razor and depilatory creams (Veet® Hair Removal). Acoustic coupling gel (Guangdong University of Technology, China) was applied. Two- dimensional standard parasternal long-axis views were applied first to acquire the left atrial diameter (LAD) at the level of the aortic valve in M-mode tracing mode. Then, standard parasternal short-axis views at the level of the papillary muscle were applied by rotating the probe ninety degrees clockwise to measure end-systolic interventricular septum thickness (IVSs), end-diastolic interventricular septum thickness (IVSd), end-systolic left ventricular posterior wall (LVPWs), end-diastolic left ventricular posterior wall (LVPWd), heart rhythm (HR), left ventricular end systolic diameter (LVESD), left ventricular end diastolic diameter (LVEDD), left ventricular end systolic volume (LVESV), left ventricular end diastolic volume (LVEDV), left ventricular ejection fraction (LVEF), left ventricular fractional shortening (LVFS), and stroke volume (SV).

### Electrophysiology and AF Vulnerability Studies

Animals were anesthetized with 1.4 g/kg urethane (i.p.) and mechanically ventilated. Electrogram morphology was analyzed before AF vulnerability studies. The P-wave duration, PR interval and the ratio of P-wave duration/ PR interval (P/PR) were measured. Transesophageal atrial burst pacing was implemented to test AF vulnerability, as described previously (Qiu et al., 2018,a,b). A total of three times pacing was performed for each animal. AF was defined as positive when the duration time of each induced AF was over 5 s. Inducibility and duration time of AF were recorded. In brief, an electrode catheter (four-French, St. Jude Medical Inc., USA) was placed in the esophagus to record the esophageal electrocardiogram and to induce AF. The position of the catheter was adjusted by the height and direction of atrial waves in the esophageal electrocardiogram and determined when clearer, higher, and bi-directional atrial waves were collected. Burst pacing was delivered by a stimulator after measuring the pacing threshold. The scheme of stimuli was set up at a two-fold threshold, with a cycle length of 20 ms, and a pulse width of 5 ms, with a total duration of 30-s.

### Biochemical Detection

Twenty-four hour urine samples for urinary protein detection were collected by using metabolic cages. Blood samples were collected from abdominal aorta by using a coagulation-promoting tube after the electrophysiological test. Serum levels of albumin (ALB), urea and creatinine (Cr) were measured by the Clinical Laboratory of Guangdong Provincial Hospital of Chinese Medicine, using an automatic biochemical analyzer (Hitachi 7180). Urine protein was estimated by using a Pierce BCA protein assay kit (Thermo).

### Serum Angiotensin II (Ang II) and TGFβ1 Content Assay

Serum Ang II and TGFβ1 were measured by using ELISA kits following the producer's instructions. The Ang-II assay kit was from Cusabio (ref. CSB-E04494r; Wuhan, China), and TGFβ1 from Abcam (ref. BMS623/3).

### Histology and Immunohistochemistry

Hearts were dewatered, embedded in paraffin, cut (3 μm thickness), and stained with Sirius red and a fast green counter stain as described previously (Zhang et al., 2014; Qiu et al., 2018,a). The collagen fiber volumes (CVF) of atria were quantified by Image J software (NIH, USA) (Rasband, 1997–2018). The proportion of Sirius red–stained areas relative to the total tissue areas (the non-staining sections in interstitial spaces were excluded from quantification) were counted as CVF. Atrial expression and distribution of Collagen type I (primary antibody: Abcam, ab34710), α-smooth muscle actin (α-SMA, CST, #19245), CTGF (Santa Cruz, sc-101586), N-cadherin (Santa Cruz, sc-59987), Cx40 (Abcam, ab183648) and Cx43 (CST, #3512) were studied using an immunohistochemistry method, as described previously (Qiu et al., 2018,a,b). Sections were incubated with a HRP antibody for 30 min at 37°C. A DAB chromogenic reagent kit (Maxim Biological Technology Development Co., LTD, Fuzhou, China) was used for coloration. Quantification was calculated by Image-Pro Plus 6.0 (Media Cybernetics, USA).

### Western Blot Analysis

The left atrium (LA) tissue was lysed in 1 mL RIPA lysis buffer containing a 1 mM PMSF and a 1% phosphatase inhibitor cocktail (Applygen Technologies Inc., Beijing, China). The protein concentration was measured by using a Pierce BCA protein assay kit (Thermo). Twenty-five to fifty micrograms of samples were separated onto 10% SDS–PAGE for protein electrophoresis and wet transferred to polyvinylidene difluoride membrane (EMD Millipore, USA). After blocking with 5% non-fat milk, membranes were incubated with a rabbit anti-rat TGFβ1 antibody (1:1000, Abcam, ab92486), phosphorylated-Smad 2 antibody (1:1000, CST, #3104), a phosphorylated-Smad 3 antibody (1:1000, CST, #9520), a α-SMA antibody (1:1000, CST, #19245), a collagen type I antibody (1:1000, abcam, ab34710), NLRP3 (1:1000, abcam, ab214185), an apoptosis-associated speck-like protein containing a carboxy-terminal CARD (ASC, 1:500, Proteintech group, 10500-1-AP), caspase-1 (1:500, Proteintech group, 22915-1-AP), IL-1β(1:1000, abcam, ab9787), IL-18 (1:1000, abcam, ab191860), Rac-1 (1:1000, abcam, ab33186), Cx40 (1:1000, Abcam, ab183648), total Cx43 (1:1000, CST, #3512); phosphorylated-Cx43 (1:1000, CST, #3511), a β-Tubulin antibody (1:1000, CST, #2146), and a GAPDH antibody (1:1000, CST, #5174), respectively, at 4°C overnight with gentle shaking. Membranes were washed three times and then incubated with a HRP-linked secondary antibody (1:3000, CST, #7074). The immunoreactivity was detected by using an enhanced chemiluminescence reagent (WBKLS0500, EMD Millipore), and then quantified by Image Lab 5.2.1 software (Bio-Rad, Hercules, USA).

### Statistical Analysis

Data were expressed as mean ± standard deviation or median (2.5–97.5 percentile) and were analyzed using the SPSS version 19. The student's independent *t*-test or Mann-Whitney *U*-test was used to analyze the difference between groups after a normal verification and homogeneity of variances analysis. The histograms were produced using the GraphPad Prism version 5 (San Diego, USA). A *P* < 0.05 was accepted as significant.

## Results

### Physical and Biochemical Characteristics

Three months post-surgery, body weight and ALB were significantly decreased in the CKD group compared to the sham group (Table 1). In addition, urea and Cr were significantly increased in the CKD group compared to the sham group. However, 24 h urine volume, urine protein concentration, and 24 h urine protein, were not statistically significant between groups.

**Table 1:** Physical and Biochemical characteristics.

**Parameters**	**Sham (*n* = 5)**	**CKD (*n* = 10)**	***P*-values**
Body weight, g	566.50 ± 48.00	501.13 ± 28.28	0.001
Urine volume, mL	19.4 ± 12.97	19.13 ± 5.46	0.696
Alb, g/L	50.12 ± 3.14	46.41 ± 1.81	0.004
Urea, mmol/L	8.74 ± 0.29	11.55 ± 0.33	0.000
Cr, umol/L	40.60 ± 1.57	58.40 ± 2.51	0.001
24 h Urine protein, g	0.85 ± 0.55	0.93 ± 0.36	0.691
ALT, U/L	59.60 ± 29.37	42.92 ± 19.29	0.182
AST, U/L	170.20 ± 60.14	172.67 ± 67.03	0.944
ALT/AST	3.04 ± 0.80	4.18 ± 1.22	0.075

*Alb, albumin; Cr, creatinine; ALT, alanine transaminase; AST, aspartate transaminase. n = 5 and 10 in Sham and CKD group, respectively*.

### Echocardiographic Parameters

At the baseline, no significant differences were found in any of the measured parameters between groups (Table 2). Three months after surgery, LAD, IVSs, IVSd, LVPWs, LVPWd were significantly increased in the CKD group compared with the sham group. However, no significant differences in HR, LVESD, LVEDD, LVESV, LVEDV, LVEF, LVFS, and SV were found between groups.

**Table 2:** Echocardiography.

**Parameters**	**Baseline**			**Three months post-surgery**		
	**Sham (*n* = 4)**	**CKD (*n* = 10)**	***P*-values**	**Sham (*n* = 4)**	**CKD (*n* = 10)**	***P*-values**
HR	386.00 ± 11.43	385.50 ± 17.44	0.959	363.99 ± 11.43	373.77 ± 13.47	0.227
LVESD, mm	4.37 ± 0.13	4.34 ± 0.20	0.814	6.09 ± 0.50	5.65 ± 0.82	0.343
LVEDD, mm	7.35 ± 0.22	7.32 ± 0.20	0.820	9.59 ± 0.39	9.06 ± 0.62	0.146
LVESV, μL	94.87 ± 4.29	96.19 ± 9.42	0.795	188.02 ± 35.32	161.39 ± 52.54	0.375
LVEDV, μL	298.45 ± 3.49	296.44 ± 7.64	0.628	515.41 ± 46.07	457.13 ± 68.34	0.147
SV, μL	199.78 ± 7.60	197.06 ± 5.82	0.479	327.39 ± 13.23	295.74 ± 36.46	0.123
LVEF, %	68.56 ± 5.62	66.86 ± 7.73	0.699	63.75 ± 3.72	65.33 ± 7.74	0.708
LVFS, %	39.62 ± 4.33	38.56 ± 5.94	0.754	36.52 ± 2.77	37.85 ± 5.86	0.676
LAD, mm	2.61 ± 0.17	2.67 ± 0.10	0.457	3.38 ± 0.14	4.57 ± 0.55	0.000
IVSs, mm	1.32 ± 0.05	1.28 ± 0.06	0.327	1.36 ± 0.08	1.82 ± 0.22	0.002
IVSd, mm	0.62 ± 0.03	0.60 ± 0.06	0.553	0.63 ± 0.11	0.96 ± 0.16	0.003
LVPWs, mm	1.60 ± 0.04	1.60 ± 0.03	0.919	1.86 ± 0.38	2.56 ± 0.10	0.033
LVPWd, mm	0.81 ± 0.08	0.82 ± 0.07	0.795	0.88 ± 0.17	1.34 ± 0.10	0.000

*HR, heart rhythm; LVESD, left ventricular end systolic diameter; LVEDD, left ventricular end diastolic diameter; LVESV, left ventricular end systolic volume; LVEDV, left ventricular end diastolic volume; SV, stroke volume; LVEF, left ventricular ejection fraction; LVFS, left ventricular fractional shortening; LAD, left atrial diameter; IVSs, end-systolic interventricular septum thickness; IVSd, end-diastolic interventricular septum thickness; LVPWs, end-systolic left ventricular posterior wall; LVPWd, end-diastolic left ventricular posterior wall*.

### Serum Levels of TGFβ1 and Ang II

Serum concentrations of TGFβ1 and Ang II were significantly increased in the CKD group compared with the sham group (Table 3).

**Table 3:** Serum levels of TGFβ1 and Ang II.

**Parameters**	**Sham (*n* = 5)**	**CKD (*n* = 10)**	***P*-values**
TGFβ1, pg/mL	43.96 ± 33.58	138.03 ± 41.20	0.011
Ang II, pg/mL	3.21 ± 1.75	9.83 ± 7.38	0.003

*TGFβ1, transforming growth factor-β1; Ang II, angiotensin II*.

### AF Inducibility and the Changes in Electrogram Morphology

**Figure 1:**
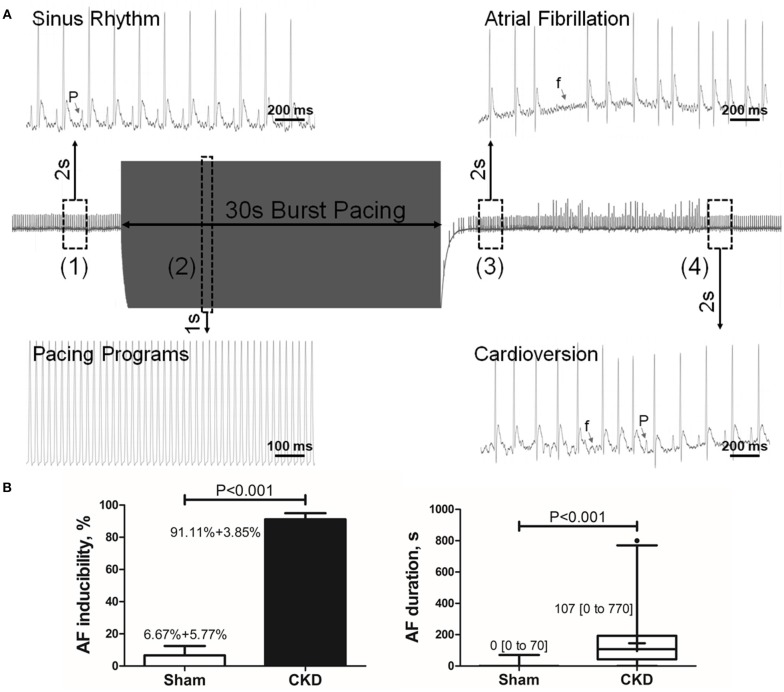
Inducible AF by transesophageal atrial pacing. **(A)** Schematic diagram of AF induction. AF was induced by a 30-s burst pacing protocol at a 20-ms cycle length, 5-ms pulse width, and 50-h frequency. AF was considered as positive if an episode of rapid, quivering atrial beating with an irregular heart rhythm lasting over 5-s, was induced. (1) Before burst pacing, P-waves are clearly distinguishable with a regular heart rhythm; (2) Burst stimuli were delivered; (3) After burst pacing, P-waves were disappeared and replaced by rapid, quivering, and sawtooth-like waves (the f-waves) with an irregular heart rhythm, suggesting an episode of AF; (4) f-waves are dispelled by P-waves, representing the termination of AF and the recovery of sinus rhythm. **(B)** AF inducibility and duration. Data show that CKD results in significant higher AF inducibility and longer AF duration compared with the sham group. AF was induced three times for acquiring the standard deviation of AF inducibility. AF inducibility is shown as mean + SD and the duration is shown as median [2.5–97.5 percentile]. *n* = 10 and 15 in Sham and CKD group, respectively. AF, atrial fibrillation; SR, sinus rhythm.

**Figure 2:**
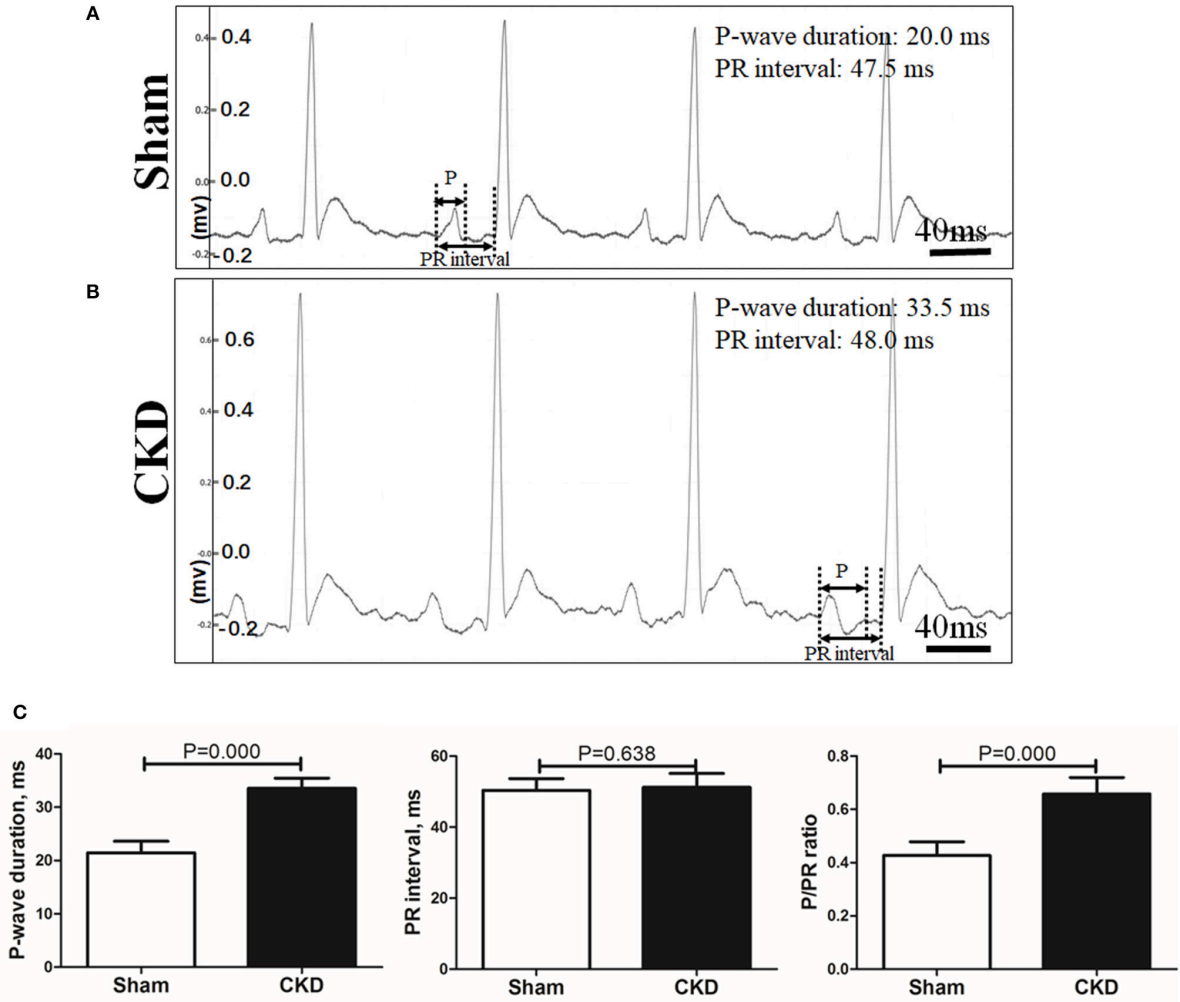
Electrogram morphology. P-wave duration and PR interval were measured from an electrogram (ECG, lead II) before burst pacing. **(A,B)**. Representative ECG recordings from the sham and the CKD group. In the sham group, P-waves are unidirectional whereas P-waves are biphasic and prolonged in CKD group. **(C)** Data suggested that P-wave duration and the ratio of P-wave duration/PR interval (P/PR) are significantly increased in the CKD group than those in the sham group while PR intervals revealed no difference. Data are shown as mean + SD. *n* = 8 and 9 in Sham and CKD group, respectively.

A representative procedure of AF inducibility is shown in Figure 1A. AF inducibility and duration times significantly increased in the CKD group compared with the sham group (Figure 1B). The morphology and duration of the P-waves were also analyzed as shown in Figure 2. The morphology of the P-wave in the inferior lead II showed biphasic waveforms in CKD rats compared with the sham group rats (Figures 2A,B). The results showed that the duration of the P-wave and the ratio of P/PR in the electrogram increased significantly in CKD rats compared with the sham group rats (Figure 2C).

### Atrial Interstitial Fibrosis

**Figure 3:**
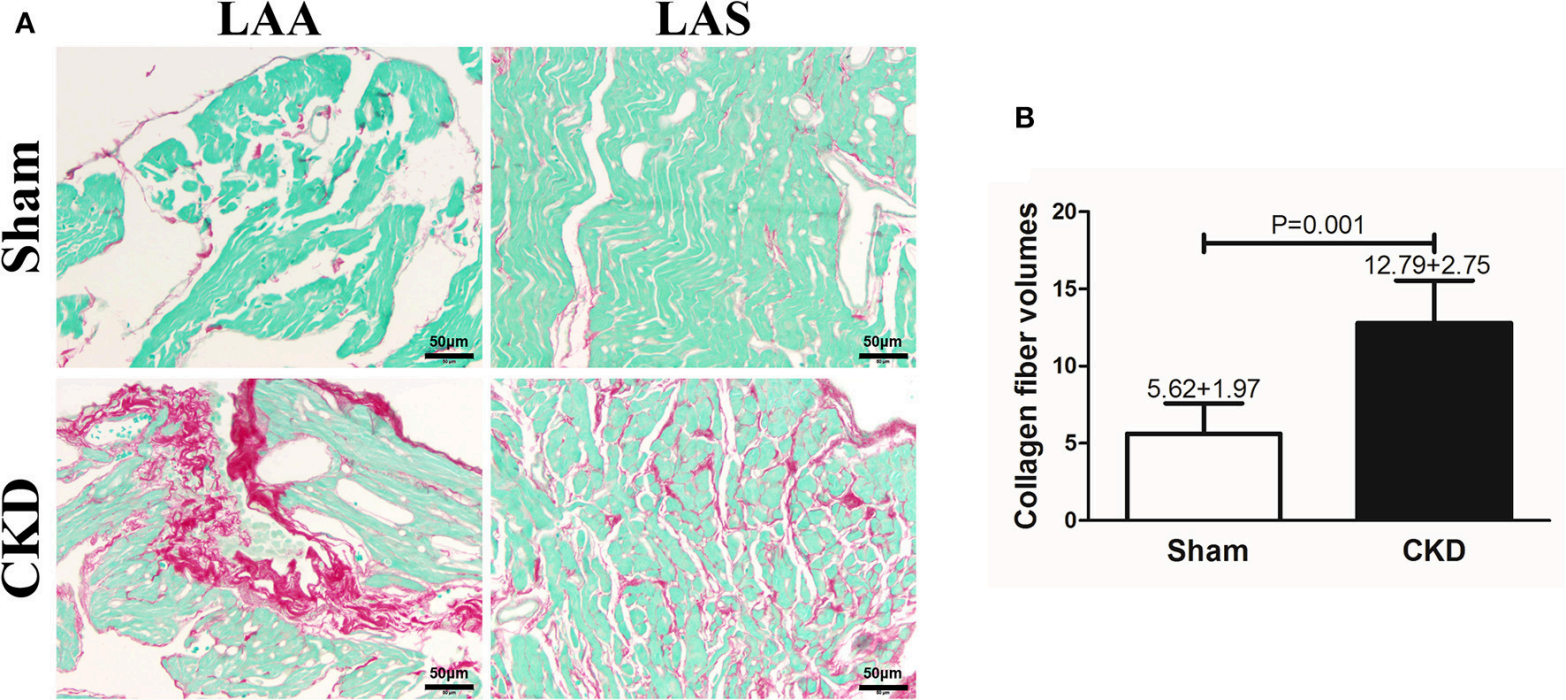
Atrial interstitial fibrosis by Sirius red-fast green counter stain. (**A)** Microscopy ( × 200) shows that atrial interstitial fibrosis was dyed red and myocytes dyed green. Atrial myocytes seem to be more diffused and hypertrophic in CKD rats compared with the sham group. **(B)** A marked increase in atrial interstitial fibrosis was found in CKD rats compared with the sham group. Data are shown as mean + SD. *n* = 5 and 6 in Sham and CKD group, respectively. Scale bar = 50 μm. LAA, left atrial appendage; LAS, left atrial septum.

Fibrosis was dyed red by Sirius red and Sirius red staining was used to assess atrial interstitial fibrosis. CVFs were calculated as the percentage of the red-stained area to the whole area except for the blank zone in Image J. As shown in Supplementary Material 1, atrial interstitial fibrosis can be accurately identified by using Image J. Representative staining images are shown in Figure 3A. The results suggested that fibrosis in both the left atrial appendage (LAA) and the left atrial septum (LAS) was significantly increased in the CKD group compared with the sham group (*P* = 0.001, Figure 3B.

### Effects of CKD on Atrial Protein Expression

**Figure 4:**
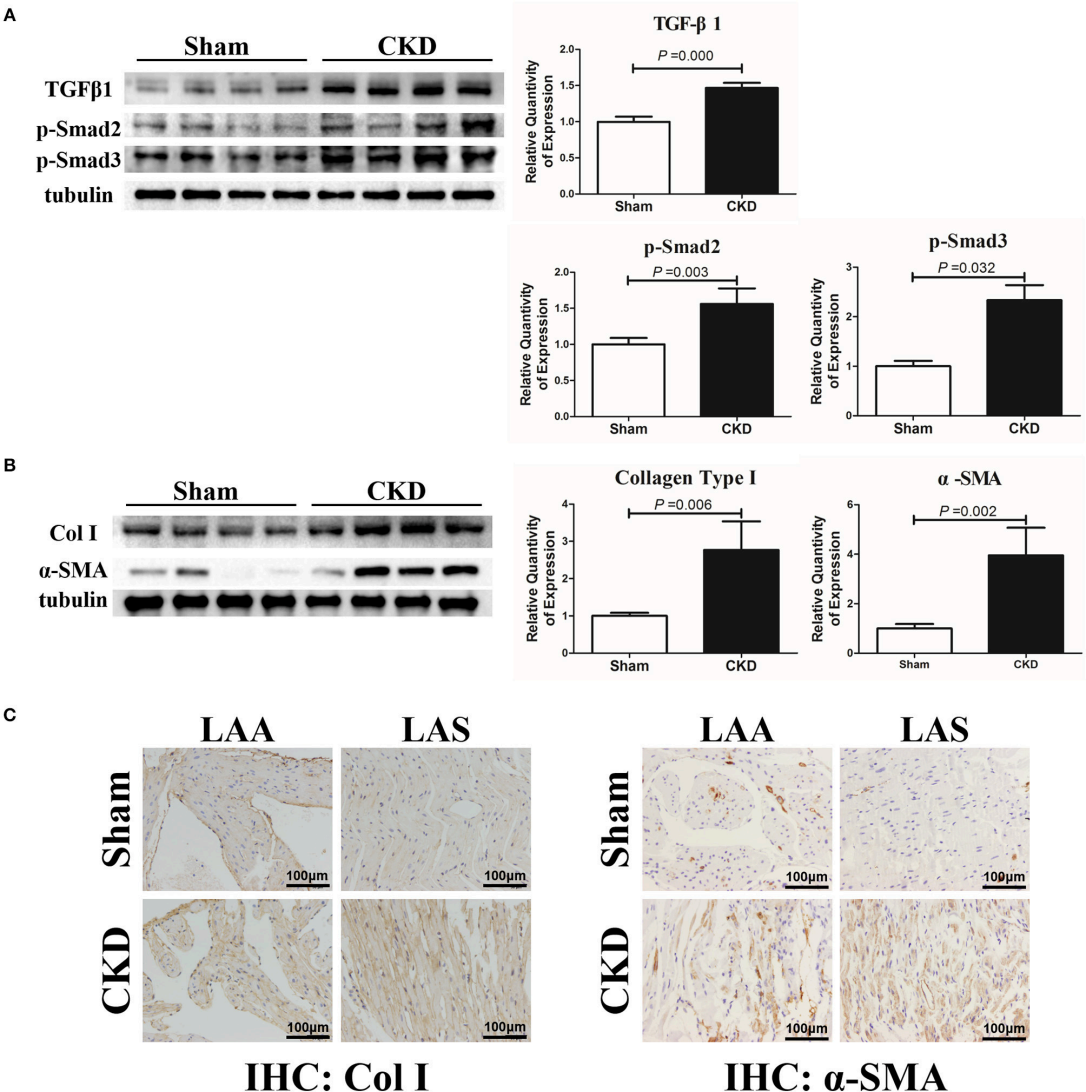
TGFβ1/Smad2/3 signal pathway is activated in CKD rats. (**A)** Expression of TGFβ1, p-Smad2, and p-Smad2, *n* = 4 per group; **(B)** Expression of collagen type I (Col I), α-SMA from fresh left atria tissues, *n* = 4 per group; **(C)** Immunohistochemistry of Col I and α-SMA, *n* = 6 per group, magnifications × 200, scale bar = 100 μm. LAA, left atrial appendage; LAS, left atrial septum.

The protein expressions of TGFβ1, p-Smad2, p-Smad3, α-SMA, and collagen type I were markedly upregulated in the CKD group compared with the sham group (Figures 4A,B). Immunohistochemistry indicated a significant deposition of α-SMA (CKD vs. Sham: 0.20 ± 0.03 vs. 0.15 ± 0.02, *n* = 6 per group, *P* = 0.005) and collagen type I (0.15 ± 0.02 vs. 0.11 ± 0.01, *n* = 6 per group, *P* = 0.000) in the CKD group compared with the sham group (Figure 4C).

**Figure 5:**
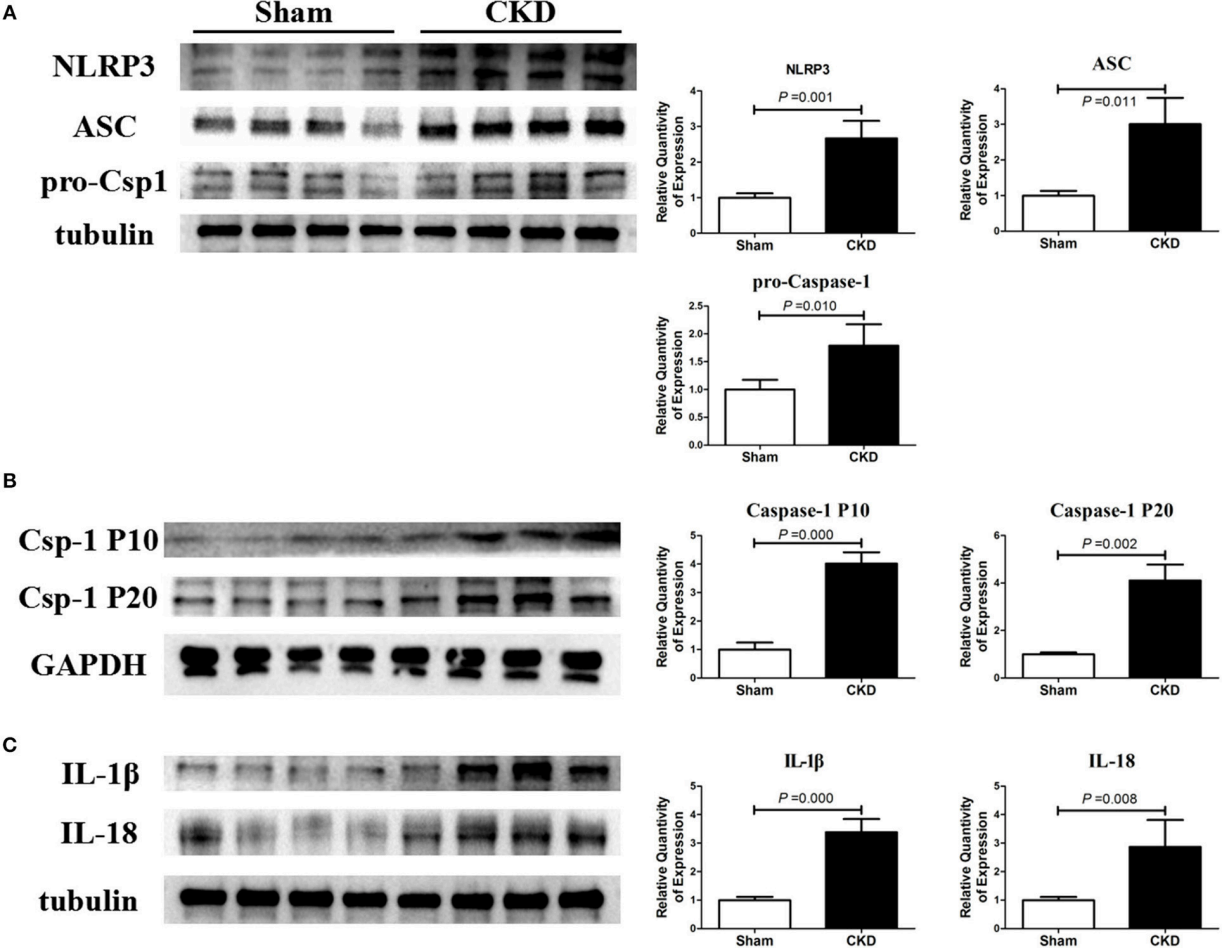
NLRP3 inflammasome/caspase-1/IL-1β and IL18 axis is activated in CKD rats. (**A)** Protein expressions of NLRP3, ASC, and pro-caspase-1 (pro-csp1); **(B)** Protein expressions of the matured caspase-1, the P20 and P10; **(C)** Protein expressions of the matured IL-1β and IL-18. Tissue was collected from the left atrium. *n* = 4 per group. ASC, apoptosis-associated speck-like protein containing a carboxy-terminal CARD; Csp-1 P10, active caspase-1 (10 kDa subunit); Csp-1 P20, active caspase-1 (20 kDa subunit); IL-1β, interleukin-1β; IL-18, interleukin-18; NLRP3, NLR (nucleotide-binding domain leucine-rich repeat-containing receptor) pyrin domain-containing protein 3; pro-csp1, pro-caspase-1.

The protein expressions of NLRP3, ASC and procaspase-1 were significantly higher in the CKD group compared with the sham group, suggesting an upregulated NLRP3 inflammasome (Figure 5A). Higher protein expression of cleaved caspase-1 (P10 and P20), IL-1β, and IL18 were also found in the CKD group compared with the sham group (Figures 5B,C).

**Figure 6:**
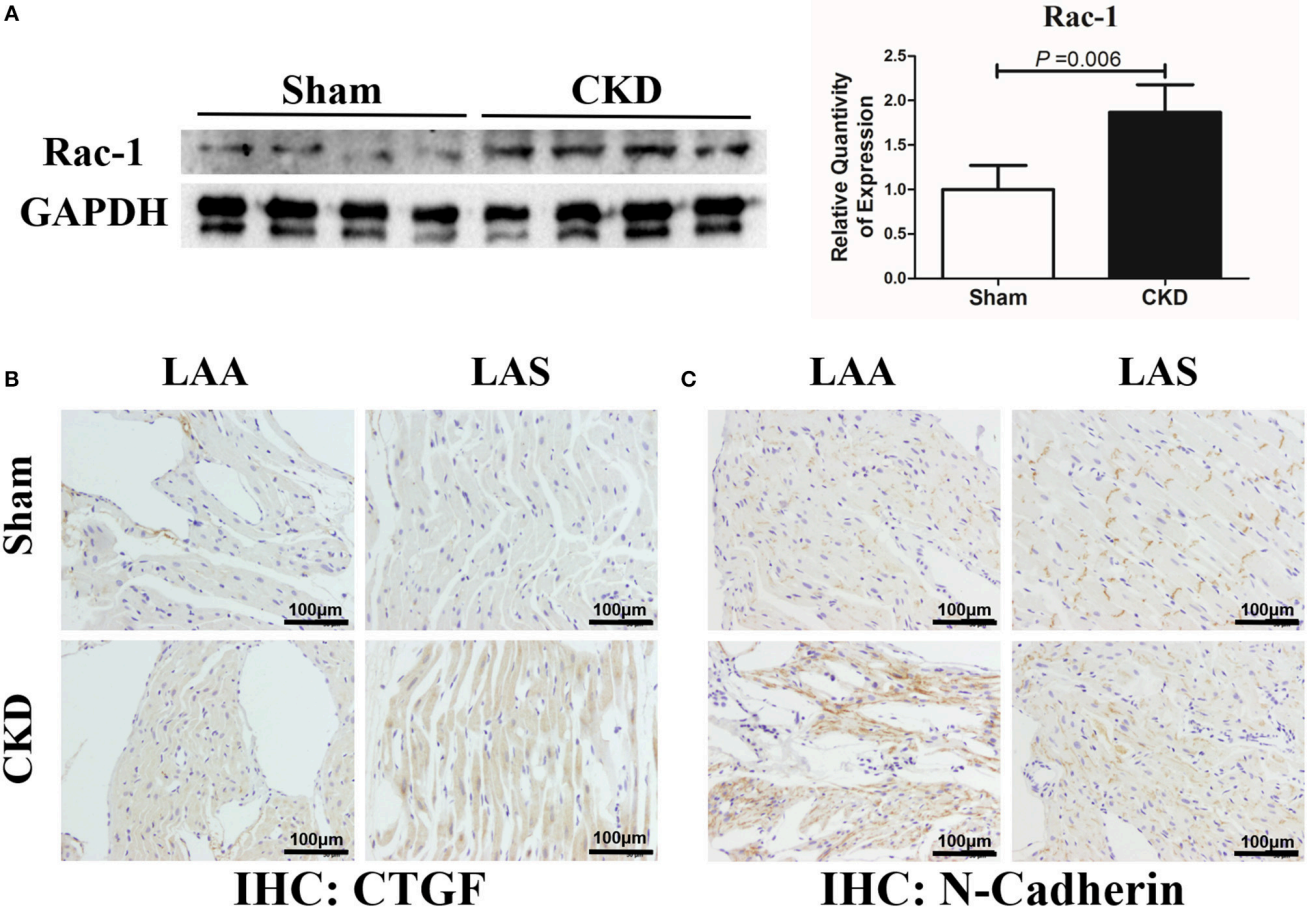
Increased Rac-1, CTGF and N-Cadherin expression in LA of rats with CKD. **(A)** Protein amount of Rac-1, *n* = 4 per group; **(B,C)**. Immunohistochemistry of CTGF and N-cadherin, *n* = 6 per group, magnifications × 200, scale bar = 100 μm. CTGF, connective tissue growth factor; Rac1, Ras-related C3 botulinum toxin substrate 1; LAA, left atrial appendage; LAS, left atrial septum.

The protein expression of Rac-1 by western blotting (Figure 6A), CTGF (0.06 ± 0.01 vs. 0.14 ± 0.04, *n* = 6 per group, *P* = 0.001) and N-cadherin (0.08 ± 0.01 vs. 0.12 ± 0.01, *n* = 6 per group, *P* = 0.000) by immunohistochemistry (Figures 6B, C) were significantly increased in the CKD group compared with the sham group.

**Figure 7:**
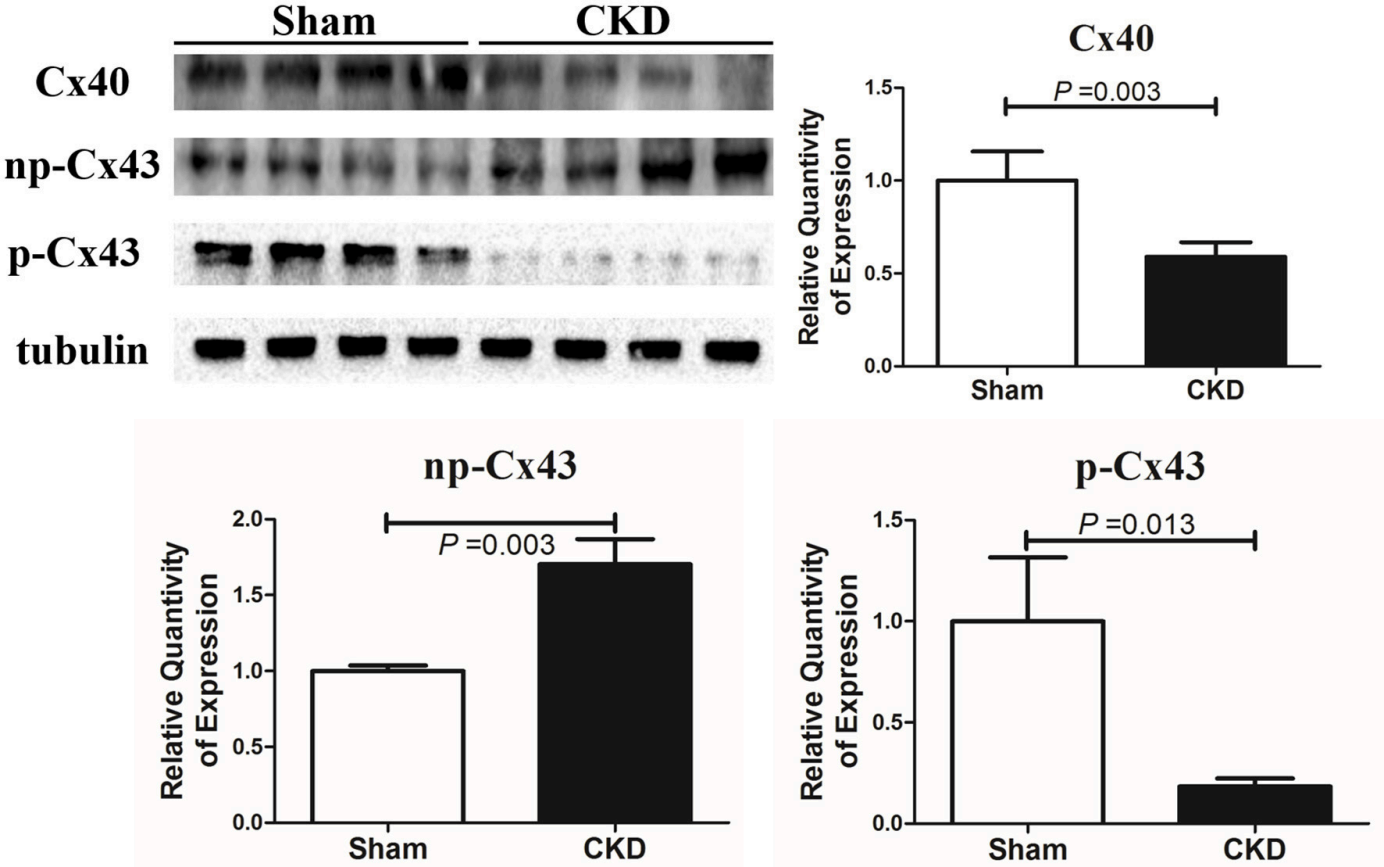
Protein amount of Cx40 and Cx43 by western blot. Shown are protein expressions of Cx40, non-phosphorylated-Cx43 (np-Cx43) and phosphorylated-Cx43 (p-Cx43). These results show that CKD resulted in a decrease in Cx40 and p-Cx43 and an increase in np-Cx43. *n* = 4 per group.

**Figure 8:**
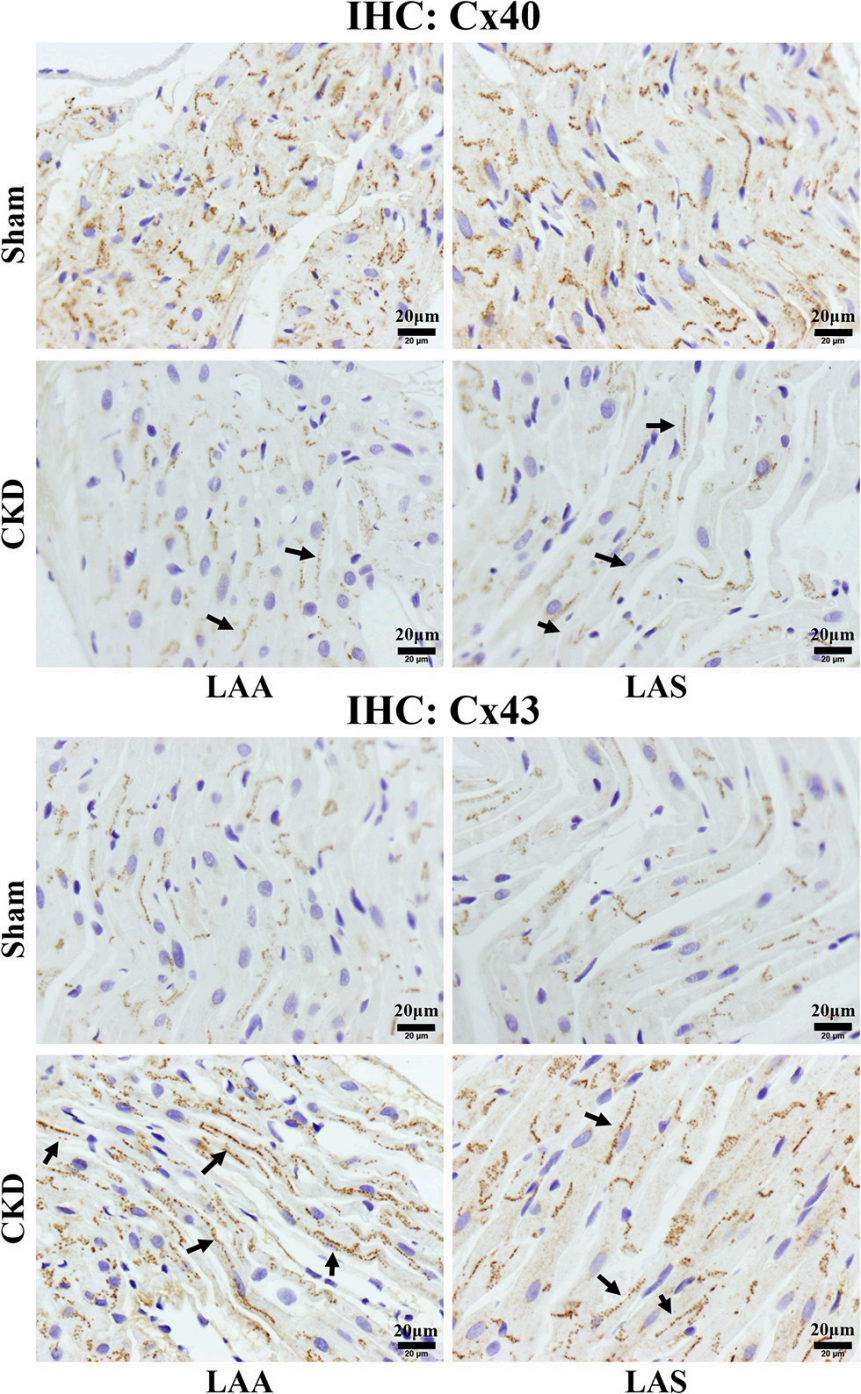
Immunohistochemistry staining of Cx40 and Cx43. The protein level of Cx40 was lower but Cx43 higher in the CKD group compared with the sham group in both LAA and LAS (data are shown in the results section). Additionally, the distribution of both proteins are more lateralized in the CKD group than those in the sham group. Magnifications × 400, scale bar = 20 μm. LAA, left atrial appendage; LAS, left atrial septum. (→) indicates lateralized Cx40 or Cx43.

Western blot analysis showed that the total Cx43 was increased significantly whereas Cx40 and p-Cx43 were decreased significantly in the CKD group compared with the sham group (Figure 7). Immunohistochemistry showed that both Cx40 and Cx43 became more lateralized with a significantly increased protein content of total Cx43 (0.01 ± 0.01 in CKD vs. 0.15 ± 0.01 in Sham, *n* = 6 per group, *P* = 0.000) and a significantly decreased protein content of Cx40 (0.07 ± 0.01 in CKD vs. 0.12 ± 0.01 in Sham, *n* = 6 per group, *P* = 0.000) in the CKD group compared with the sham group (Figure 8).

## Discussion

The present study provides evidence for the pathophysiological changes of CKD-enhanced AF. CKD resulted in the left atrial enlargement, interstitial fibrosis, and inducible AF, which may be related to the activation of TGFβ1/Smad2/3 and the NLRP3 inflammasome signaling as well as connexins remodeling. Although the present study was not the first to establish a model of AF associated with CKD in rats, it was the first to investigate the changes of TGFβ1 related-downstream mediators, NLRP3 inflammasome and connexins remodeling in a CKD setting.

In this study, CKD was established by the 5/6 nephrectomy in rats. It is an effective, feasible, and direct method based on the biochemical characteristics and our previous study (Zeng et al., 2016). The echocardiographic results indicated that CKD caused a hypertrophic cardiomyopathy and LA enlargement, without ventricular dysfunction. AF was induced by transesophageal atrial burst pacing, a classic method that was used to assess the vulnerability to AF (Rahmutula et al., 2013; Qiu et al., 2018,a,b). Other methods to induce AF is also be available by implanting a pacemaker for some weeks (Harada et al., 2012). Our results showed that the inducibility of AF was 91.11%. This result is much higher than that in our previous study on an animal model of heart failure in the same stimulus protocol (Qiu et al., 2018,a,b). Additionally, our results found that P-waves were extended and biphasic; and the ratio of P/PR was increased in CKD rats. Clinically, these changes can usually be found in Bayés Syndrome, which suggests an advanced interatrial block that mainly results from atrial interstitial fibrosis or connexins remodeling and is highly suggestive of AF in a short-term follow-up (Baranchuk et al., 2018). The ratio of P/PR, also called the Macruz index, provides a clue for heterogeneous electrical conduction (Human and Snyman, 1963).

The progressive deposition of an extracellular matrix creates a structural milieu contributing to the onset and recurrence of AF, by interrupting normal cardiac muscle bundles in their longitudinal orientation. TGFβ1, as an important mediator of atrial fibrosis and AF, is one of the major downstream mediators of Ang II and has been fully elucidated in multiple diseases including heart failure, myocardial infarction, and hypertension, etc., in humans and in animals (Everett and Olgin, 2007; Corradi et al., 2008). TGFβ1 can mediate the phosphorylation of Smad2/3, regulating the profibrotic gene expression. Additionally, Ang II can induce the activation of both TGFβ1/Smads pathway and oxidative stress, acting to produce fibrotic remodeling (Yeh et al., 2011). NAPDH oxidase mediated-oxidative stress plays a role in the pathogenesis of CKD induced-AF which has previously been demonstrated (Fukunaga et al., 2012). However, the critical links between TGFβ1 and atrial fibrosis in CKD remain unclear. Our results revealed that the upregulated TGFβ1 was associated with an activated p-Smad2/3, identifying one of the downstream mediates of TGFβ1 in CKD-induced AF.

CTGF is also a downstream mediator of TGFβ1 and is of vital importance in the regulation of cell mitosis, proliferation and differentiation of fibroblasts and collagen synthesis (Booth and Bishop, 2010). A previous study showed that the serum TGFβ1 can promote CTGF synthesis and induce left atrial enlargement and remodeling, involved in the pathogenesis of AF (Lin et al., 2015). Most importantly, Ang II can also induce fibrosis via CTGF (Rupérez et al., 2003). Our results suggest that CTGF may play a role in CKD induced-atrial fibrosis and AF, due to the increased serum TGFβ1 and Ang II. As CTGF is mainly activated in the final phase of fibrosis, rather than targets in the initial phase like TGFβ1, therefore suggesting that CTGF may be more valuable to intervene with reversing fibrotic remodeling because it has fewer side effects (Rupérez et al., 2003). Further research is needed to investigate its specific mechanism and potential implication.

NLRP3 inflammasome/caspase-1/IL-1β and IL18 axis forms an innate immune response and plays a role in the pathogenesis of fibrosis in multiple organs including the heart, lung, kidney and the liver (Bracey et al., 2014; Rimessi et al., 2015; Cai et al., 2016; Gong et al., 2016; Stout-Delgado et al., 2016; Chin et al., 2017; Romero et al., 2017; Yao et al., 2018). Ang II was found to stimulate the activation of the NLRP3 inflammasome (Cai et al., 2016). Inversely, NLRP3 depletion can inhibit Ang II-induced fibrosis (Cai et al., 2016), block the differentiation of myofibroblasts and reduce the activation of R-Smad in response to TGFβ1 stimulation (Bracey et al., 2014). These data suggest that the NLRP3 inflammasome is critical in the regulation of Ang II-induced fibrosis. Additionally, CKD can also activate the NLRP3 inflammasome/IL-1β/IL-18 axis, contributing to ventricular contractile dysfunction (Chin et al., 2017). However, whether CKD can provoke the activation of the NLRP3 inflammasome and urge inflammatory cascade response in the atrium, remains unknown. Our results showed that CKD increased the activation of the axis in the atrium which was accompanied with increased Ang II and TGFβ1, suggesting a potentially novel therapeutic target involved in CKD enhanced-AF.

Connexins remodeling is an important aspect of Ang II induced-ASR and AF. A previous study reported that Ang II upregulates the protein expression of CTGF via the Rac1 and N-cadherin, contributing to connexins remodeling (Adam et al., 2010). Since an elevated level of Ang II was found, we further investigated whether connexins would be influenced in a CKD setting. The present study showed that CKD increased the expression of np-Cx43, decreased the expression of Cx40 and p-Cx43, and urged the lateralized distribution of Cx43/40 in the atrium, suggesting a connexins remodeling. Normally, connexins are distributed at the adhesion point of intercalated disc, playing a crucial role in maintaining intercellular electrical conduction along the long axis of myocardial fibers. Connexins remodeling including the disorders of expression and distribution are associated with pro-arrhythmic conduction slowing and conduction heterogeneity (Akar et al., 2004), represented as biphasic P-wave and an increased ratio of P/PR in electrocardiogram. Our results showed that the connexins remodeling in the atrium was accompanied with the up-regulation of Rac-1, CTGF and N-cadherin. Rac1 is a small guanosine triphosphate binding protein, a member of the Rho GTPase superfamily of intracellular signal transducers (Satoh et al., 2006). N-cadherin mainly regulates the cell-cell adhesion in the intercalated disc, relating to the intercellular coupling function of connexins gap junction (Rucker-Martin et al., 2006). A previous study has showed that Rac1 can be activated by Ang II and TGFβ1 and then rearrange Cx43 and N-cadherin (Satoh et al., 2006; Tsai et al., 2008; Adam et al., 2010). Therefore, in agreement with the above results, our results showed that a connexins remodeling in CKD may be associated with the activation of Rac1, CTGF and N-cadherin.

Excessive atrial interstitial fibrosis, atrial structural alteration and inflammation may break the cytoskeleton network and damage the adhesion proteins that contribute to the normal organization of connexins (Giepmans, 2004; Rucker-Martin et al., 2006). The mechanical deformation of the atrial wall, by chronic hemodynamic overload or by hypertension, both of which are secondary to CKD, could induce Cx40/43 remodeling (Haefliger and Meda, 2000; Severs et al., 2008). It has been demonstrated that exposure to inflammation can reduce Cx40/43 functional expression and change gap junctions in the heart, whereas CKD causes systemic inflammation and activates TGFβ1/Smad2/3 and NLRP3 inflammasome (Sawaya et al., 2007; George et al., 2017). Therefore, taken together with our observation, connexins remodeling is one of pathological changes involved in CKD enhanced-AF.

## Limitations

Our results suggested that the TGFβ1/Smad2/3 signal pathway, the NLRP3 inflammasome and connexins remodeling are involved in CKD enhanced-ASR and AF. These pathological factors, in fact, are interactional. The NLRP3 inflammasome induced-the activation of IL-1β and IL-18 is known to be profibrotic, most importantly it can constitute a positive feedback to the activation of TGF-β1 and exacerbate connexins remodeling (Kolb et al., 2001; Luo et al., 2009; Fix et al., 2011). Inevitably, the present study has shortcomings. Firstly, the specific causes which induced the above pathological changes are unknown. CKD is always complicated with hypertension, heart failure, chronic volume overload, etc., of which alone or together can create the arrhythmogenic substrate for AF initiation. However, it is hard to constitute a model isolating the additional effects of CKD. Secondly, no interventions were taken. The potential effects of antagonist against the activation of RAAS, TGFβ1/Smad2/3, the NLRP3 inflammasome, CTGF and Rac-1 on CKD induced-AF are unknown. The effects of angiotensin converting enzyme inhibitor and angiotensin receptor blocker against Ang II are worthy of further studies as suggested by the present findings. Thirdly, the influences of different periods and different degrees of kidney dysfunction on ASR and AF are not involved. The reason why we chose a CKD rat model of 3 months post-5/6 nephrectomy to investigate the potentially pathological changes was meant to constitute a more stable CKD model based on previous data, and to avoid acute factors, unstable/incomplete kidney failure, and insufficient duration time. Fourthly, the impacts of CKD on atrial electrical remodeling and the functional changes of ion channels were not involved in the present study. Although the characteristics of CKD enhanced-ASR and AF are considered systemic and multifactorial, to validate these pathological changes is important to point out future directions. In short, further studies focused on pathological mechanisms and therapeutic reactions are worthy of investigation.

## Conclusions

Despite upstream therapy that is intended to stop the prevalence of AF by preventing or reversing ASR has been proposed, the prevention of CKD-related AF still has a long way to go due to the lack of a clear understanding of the pathophysiologic mechanisms. In the present study, we investigated the potential pathogenesis of AF in a rat model of CKD established 3 months post-5/6 nephrectomy. Our results showed that CKD led to the left atrial enlargement, increased the vulnerability to AF with prolonged and biphasic P-wave. Pathologically, CKD caused severe atrial interstitial fibrosis and connexins remodeling. Possible mechanisms of ASR and connexins remodeling in CKD should include the TGFβ1/Smad2/3 signaling, NLRP3 inflammasome/caspase-1/IL-1β and IL18 axis, and the changes of Rac-1, CTGF and N-cadherin. In conclusion, these findings implicate TGFβ1/Smad2/3, the NLRP3 inflammasome and connexins as potential mediators of increased AF vulnerability in CKD.

## New and Noteworthy

The present study reports that CKD causes an atrial structural remodeling and inducible AF in a CKD rat model, 3 months post-5/6 nephrectomy. These pathophysiological changes are linked to the activation of TGFβ1/Smad2/3 signaling, NLRP3 inflammasome and connexins remodeling. The present study may produce an effect on the upstream therapy for CKD-enhanced AF.

## Author Contributions

The conception and design were proposed by HQ, CZ, WJ, YW, ZL, QL, ZX, XL, and HW. Animal and molecular biology experiments were mainly finished by HQ, CJ, and WL. Paper was drafted by HQ and reviewed by CZ and WJ.

### Conflict of Interest Statement

The authors declare that the research was conducted in the absence of any commercial or financial relationships that could be construed as a potential conflict of interest.

## Abbreviations

AF: atrial fibrillation
ALB: albumin
Ang II: angiotensin II
ASC: apoptosis-associated speck-like protein containing a carboxy-terminal CARD
ASR: atrial structural remodeling
α-SMA: α-smooth muscle actin
CKD: chronic kidney disease
CKI: cardiomyocyte-specific knock-in murine model
CM: cardiomyocyte
Cr: creatinine
CTGF: connective tissue growth factor
Cx: connexin
HR: heart rhythm
IL-1β: interleukin-1β
IL-18: interleukin-18
IVSd: end-diastolic interventricular septum thickness
IVSs: end-systolic interventricular septum thickness
LA: left atrium
LAA: left atrial appendage
LAD: left atrial diameter
LAS: left atrial septum
LVEDD: left ventricular end diastolic diameter
LVEDV: left ventricular end diastolic volume
LVEF: left ventricular ejection fraction
LVESD: left ventricular end systolic diameter
LVESV: Left ventricular end systolic volume
LVFS: left ventricular fractional shortening
LVPWd: end-diastolic left ventricular posterior wall
LVPWs: end-systolic left ventricular posterior wall
NADPH: nicotinamide adenine dinucleotide phosphate
NLR: nucleotide-binding domain leucine-rich repeat-containing receptor
NLRP3: NLR (nucleotide-binding domain leucine-rich repeat-containing receptor) pyrin domain-containing protein 3
P/PR: the ratio of P-wave duration/ PR interval
RAAS: Rennin-Angiotensin-Aldosterone System
SV: stroke volume
TGFβ1: transforming growth factor-β1.
