# Supplementary material for: Chronic Kidney Disease Increases Atrial Fibrillation Inducibility: Involvement of Inflammation, Atrial Fibrosis, and Connexins
Source: Front Physiol. 2018 Dec 4;9:1726. doi: 10.3389/fphys.2018.01726 (PMC6288485; doi:10.3389/fphys.2018.01726)
Supplement: Supplementary file 1 [file Data_Sheet_1.docx]

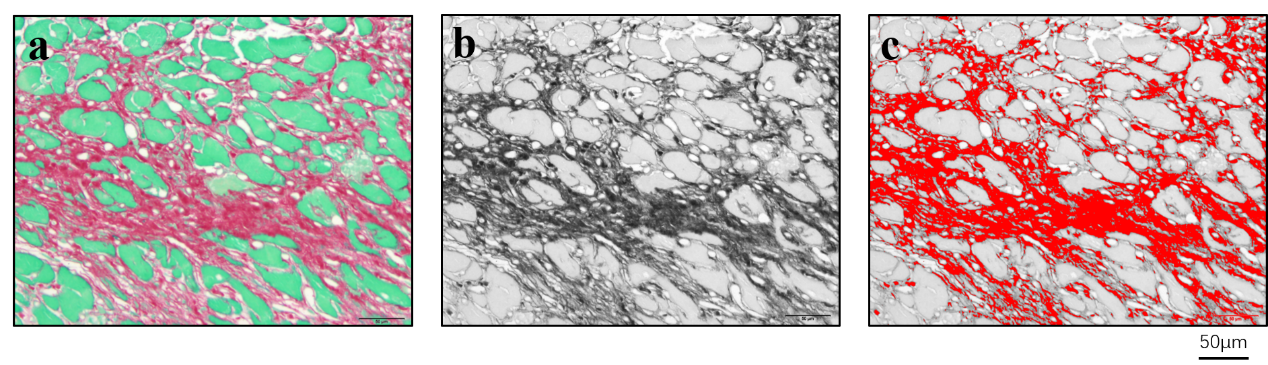


**Supplementary material 1. Sirius red and fast green counter stain.** Atrial collagen fiber volumes were quantified by using Image J software (NIH, USA) (27). Atrial interstitial fibers can be accurately identified by Image J as shown in a-b-c. **a.** the original picture of sirius red and fast green counter stain; atrial interstitial fibers were dyed red and atrial myocytes were dyed green. **b.** the identified (black) areas by Image J. **c.** the calculated areas. Operation procedures of Image J are briefly introduced below. (1) Firstly, open the image through Image J; (2) Click “Analyze\ Set Scale” to set the conversion relation, select "Global" and click “OK” to complete the setting; (3) Click “Image \ Color \ Split Channels” to well recognize the red-staining areas which will then be recognized as black-dyed areas. (4) Click “Image \ Adjust \ Threshold” to manually adjust the range of the fibers area till the black-dyed areas can be exactly covered. (5) Click “Analyze \ Set Measurements” to display the analyzed content in an opening window (“Area”, “Area fraction”, “Limit to threshold”, and “Display label” were chosen), then click “Analyze \ Measure” to complete the calculation. Data can be exported via “File\ Save As”.
